# Evaluation of target search efficiency for neurons during developmental growth

**DOI:** 10.1186/1471-2202-13-S1-P75

**Published:** 2012-07-16

**Authors:** Gloria Sanin, Emily Su, Troy Shinbrot, Remus Osan

**Affiliations:** 1 Department of Mathematics and Statistics, Georgia State University, Atlanta, GA, 3030, USA; 2 Department of Biomedical Engineering, Rutgers University, Piscataway, NJ, 08854, USA; 3 Neuroscience Institute, Georgia State University, Georgia State University, Atlanta, GA, 30303, USA

## 

Spinal cord injury can lead to permanent damage of the ascending and descending neural pathways, which can in turn induce a loss of sensory feeling and motor control in the limbs, respectively. The growth of neuron can potentially result in experimental therapies, but the issue of targeting the exact regions remains problematic. While mathematical and computational research has traditionally focused on faithful characterization of the morphological properties of the neurons, less emphasis has been placed on understanding how the growth rules of the neurons translate in effective targeting of desired neural regions. To quantitatively analyze and optimize targeting strategies, we have developed side-by-side *in silico* and *in vitro* experimental studies of neuron growth, branching and pruning using chicken DRG neurons as a prototypical model system [1]. In this work we investigated how branching and pruning influence the probability of successfully connecting to neurons located at different locations away from the initiation point, under the assumption that the neuron has finite growth resources. We find out that balanced branching and pruning, and the distance to target are essential in determining the optimal growth parameters. For example, fast branching neurites that do not prune will exhaust their resources quickly, while neurites that seldom branch and prune can reach far away distances.

We consider here two extensions of this model. Since under some conditions, branching and searching for targets may not initiate until advancing axons reach an appropriate domain, we investigate neural grown models in which pruning depends on distance from the origin (α), length (β) or order of branches (γ) [2]. We find that for the first two cases (γ, β), trimming branches nearer the origin or short-length branches can significantly improve targeting of points further away, while order-dependent pruning (γ) has the opposite effect, although to a much weaker extent.

Furthermore, previous research has not investigated how the size of the targets impacts the targeting efficiency. We explore this issue in a computational model that uses a balanced way of growing the neurons. This is done by restricting the branching angles to be equal, while the neurons evolve at constant branching rates. We use optimization to compute the ideal parameters that allow a tree to have maximal success in finding targets of specified size at a set distance from the initiation point. For this idealized neural tree, this allows us to determine the how the targeting efficiencies change with parameters and to compare these results to numerical simulation where neurons can obtain more complex morphologies.
